# A Bead Aggregation Assay for Detection of Low-Affinity Protein-Protein Interactions Reveals Interactions between N-Terminal Domains of Inositol 1,4,5-Trisphosphate Receptors

**DOI:** 10.1371/journal.pone.0060609

**Published:** 2013-03-26

**Authors:** Alap P. Chavda, David L. Prole, Colin W. Taylor

**Affiliations:** Department of Pharmacology, University of Cambridge, Cambridge, United Kingdom; Tel-Aviv University, Israel

## Abstract

Interactions between proteins are a hallmark of all cellular activities. Such interactions often occur with low affinity, a feature that allows them to be rapidly reversible, but it makes them difficult to detect using conventional methods such as yeast 2-hybrid analyses, co-immunoprecipitation or analytical ultracentrifugation. We developed a simple and economical bead aggregation assay to study low-affinity interactions between proteins. By coating beads with interacting proteins, the weak interactions between many proteins are sufficient to allow stable aggregation of beads, an avidity effect. The aggregation is easily measured to allow quantification of protein-protein interactions under a variety of controlled conditions. We use this assay to demonstrate low-affinity interactions between the N-terminal domains of an intracellular Ca^2+^ channel, the type 1 inositol 1,4,5-trisphosphate receptor. This simple bead aggregation assay may have widespread application in the study of low-affinity interactions between macromolecules.

## Introduction

Interactions between protein domains, whether within or between proteins, are universal features of cellular physiology [1]–[5]. These interactions often occur with low affinity, reflecting the need for rapid changes in the interactions to facilitate dynamic regulation of cellular activities [4]–[6]. These features, the low affinity of the interactions and their regulation by changes in cytosolic environment, present considerable challenges when attempting to explore them by methods such as yeast two-hybrid (Y2H) [7], phage-display (PD) [8], immunoprecipitation (IP) [9] or analytical ultracentrifugation (AUC) [10]. Additional limitations of these methods include the need for specialized expertise (Y2H, PD) or equipment (AUC), or large amounts of material (IP, AUC), their inability to discriminate between direct and indirect interactions (IP, AUC) and the difficulty of replicating cytosolic conditions during the assay (Y2H, AUC).

Inositol 1,4,5-trisphosphate receptors (IP_3_R) are intracellular Ca^2+^ channels. They are ubiquitously expressed in animal cells and mediate release of Ca^2+^ from the endoplasmic reticulum (ER) in response to extracellular signals that stimulate formation of IP_3_ [11]. As with all ion channels, activation of IP_3_R proceeds via re-arrangements of interactions between protein domains within the oligomeric channel. For IP_3_R, these conformational changes are initiated by IP_3_ binding to the IP_3_-binding core (IBC, residues 224–604) of each of the four IP_3_R subunits, and then proceed via re-organization of intramolecular interactions between the IBC and suppressor domain (SD, residues 1–223) [12], [13]. These conformational changes are proposed to disrupt contacts between the N-terminal regions of the four subunits and to culminate in opening of the Ca^2+^-permeable pore [13]. The versatility of IP_3_-evoked Ca^2+^ signalling is increased further by the spatial organization of IP_3_R within ER membranes [14] and their association with a diverse array of additional proteins that regulate their activity [15], [16]. The importance of interactions between protein domains for regulation of IP_3_R is clear and so is the need for simple, convenient assays to address them.

In this study, we developed an economical and rapid bead aggregation assay to study low-affinity interactions between proteins. Proteins are immobilized at high density on small beads so that individually weak interactions between protein partners on different beads collectively contribute to a multivalent interaction that causes stable aggregation of the beads. The assay is simple, inexpensive and rapid, and it requires only small amounts of proteins and a standard light microscope. It allows direct interactions between proteins to be quantified even when they occur with low affinity.

## Results and Discussion

### An assay for analysis of low-affinity interactions between macromolecules

**Figure 1:**
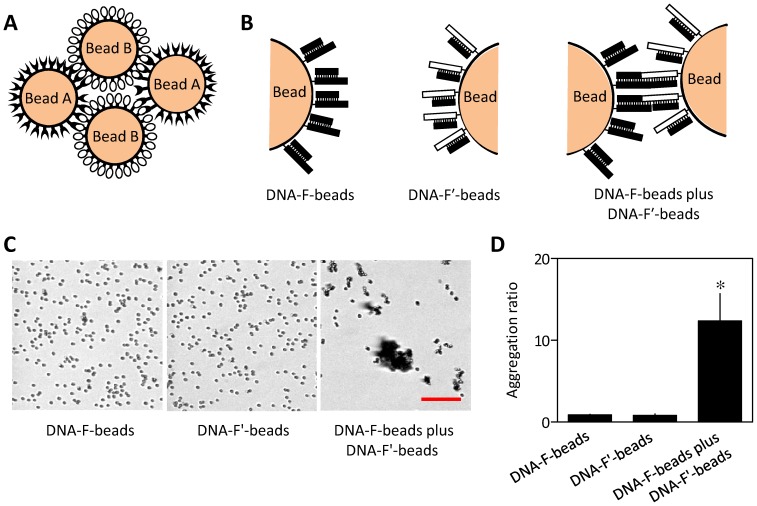
Bead aggregation assay. (A) Beads coated with interacting partners (A and B) are expected to aggregate when mixed. (B) SVA-beads coated with biotin-labelled double-stranded DNA are prepared with one population displaying a sticky end (DNA-F) and the other with a complementary sticky end (DNA-F′). Interactions between the complementary strands are expected to cause aggregation of the beads. (C) SVA beads displaying either DNA-F or DNA-F′ were prepared. Each DNA can only weakly self-dimerize with a predicted K_D_ of ∼36 mM, while a mixture of complementary DNA can form dimers with a predicted K_D_ of ∼63 fM (calculated using OligoAnalyzer 3.1, Integrated DNA Technologies). Differential interference contrast (DIC) microscopy was used to measure aggregation after incubation (25 µL, 30 min) of each population of beads alone (10 pmol of SVA sites and 20 pmol biotin-DNA) or after mixing (50∶50 ratio). The scale bar (20 µm) applies to all three images. (D) Summary results show the aggregation ratios (see Methods) as means ± SEM, from 3 independent experiments with 5 fields analyzed in each. *P = 0.017.

**Figure 2:**
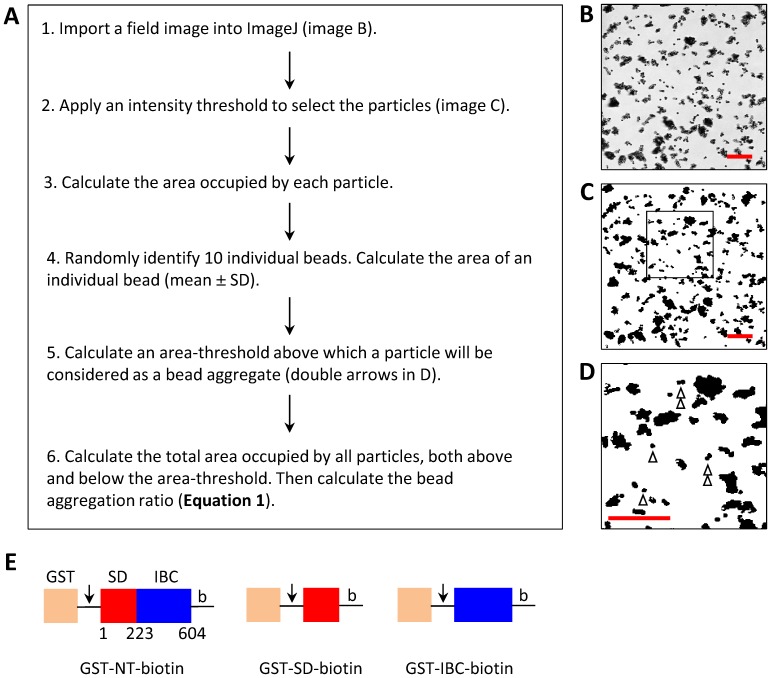
Quantification of bead aggregation. (A) The protocol used for analyzing aggregation of beads. (B–D) Differential interference contrast (DIC) images of SVA beads coated with NT-biotin showing a typical field (B), the processed image after applying an intensity-threshold (C), and an enlarged image of the box (53.6×53.6 µm) shown in C (D) with single and double arrows indicating single and double beads. Scale bars represent 20 µm in all images. (E) Cartoon representation of the constructs used. Arrows indicate the PreScission protease cleavage site and b denotes the biotinylation sequence.

We sought to develop a simple and economical assay to analyze low-affinity interactions between proteins or other weakly interacting partners. We envisaged that by immobilizing proteins on the surface of a bead, many individually weak head-to-head interactions between proteins on different beads might collectively provide an interaction with sufficient avidity to cause the beads to become stably associated (**Figure 1A**). We first tested the ability of such a bead aggregation assay to report interactions between molecules by immobilizing complementary anti-parallel DNA on two different populations of beads (**Figure 1B**). Biotinylated double-stranded DNA (DNA-F) with a sticky end was immobilized on streptavidin (SVA)-coated beads and then incubated with a second population of beads coated with biotinylated dsDNA with complementary sticky ends (dsDNA-F′) (see Methods). Beads coated with either biotin-dsDNA-F or biotin-dsDNA-F′, where DNA can only weakly self-dimerize (with a predicted equilibrium dissociation constant, K_D_, of ∼36 mM), did not aggregate. However, mixing the two populations of beads coated with complementary DNA (capable of forming dimers with a predicted K_D_ of ∼63 fM) caused them to aggregate (**Figures 1C and 1D**). This was quantified by measuring the ratio of aggregated to single beads (see Methods). The results demonstrate that interactions between complementary partners can be quantified by this simple bead aggregation assay (**Figure 1D**). The optimized protocol used to quantify aggregation of beads in subsequent assays is shown in **Figures 2A–2D**.

### Bead aggregation assay applied to analysis of interactions between N-terminal fragments of IP_3_ receptors

**Figure 3:**
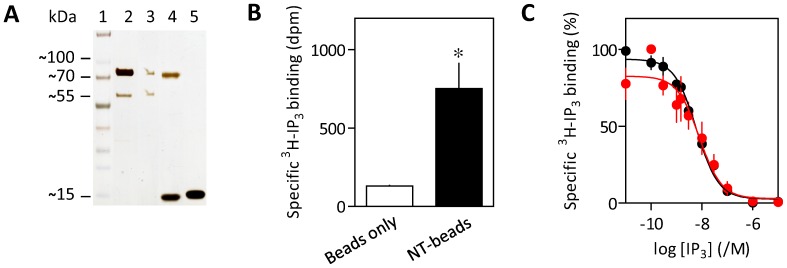
Immobilization of functional NT on beads. (A) SVA-beads (60 pmol) were incubated with NT-biotin (17 pmol) and then magnetically separated from the supernatant (see Methods). The silver-stained gel shows equivalent fractions of the input (lane 2), the supernatant (lane 3), NT-beads treated (85°C, 10 min) to release bound protein (lane 4), or similarly treated control beads (lane 5). Lane 1 shows the molecular mass markers (kDa). The 70-kDa- and 15-kDa-bands correspond to NT-biotin and SVA monomer, respectively. (B) Specific ^3^H-IP_3_ binding (0.75 nM) to control or NT-coated SVA-beads. Results (dpm, disintegrations per minute) are means ± SEM, n = 3. *P = 0.019. (C) Equilibrium-competition binding to NT-biotin (black) and NT-beads (red) with ^3^H-IP_3_ (0.75 nM). Results are means ± SEM from 3 experiments.

We next sought to apply the assay to analysis of protein-protein interactions using N-terminal domains of IP_3_R1 (**Figure 2E**). These were chosen because cytosolic domains of IP_3_R are proposed to mediate interactions between IP_3_R [17]–[19], and binding of IP_3_ to the NT (residues 1–604) initiates IP_3_R activation [12], [13]. Biotinylated NT was immobilized on SVA-coated beads (see Methods). Silver staining confirmed that NT of the appropriate size was effectively immobilized and that no residual GST-NT (predicted size ∼96 kDa) was detectable on the beads (**Figure 3A**). NT-beads selectively bound ^3^H-IP_3_ with an affinity (pK_D_ = 8.24±0.27, n = 3, where pK_D_ is the negative logarithm of the equilibrium dissociation constant, K_D_) similar to that of NT in solution (pK_D_ = 8.28±0.09, n = 3) (**Figures 3B and 3C**). These results suggest that NTs immobilized on SVA beads retain native conformations and accessible IP_3_-binding sites.

**Figure 4:**
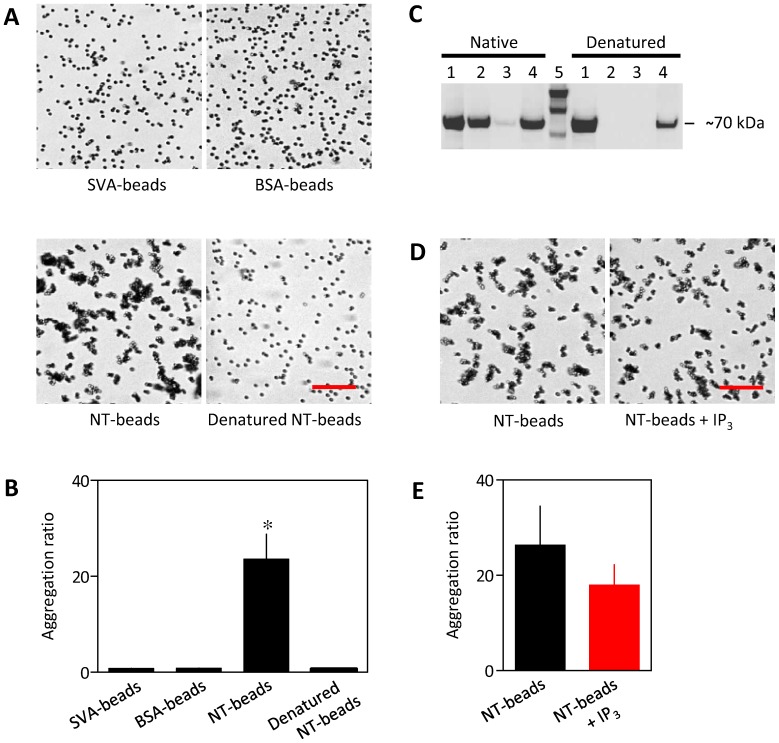
The NT of IP_3_R1 causes aggregation of beads. (A) DIC images of beads prepared as indicated. (B) Summary results show means ± SEM, n = 3. *P = 0.012 relative to SVA beads. (C) Western blot, probed using HRP-conjugated streptavidin, shows equivalent fractions of the input (20 pmol of native or denatured NT-biotin, lane 1), the supernatant (lane 2), wash (lane 3) and NT-beads treated (85°C, 10 min) to release bound protein (lane 4). Lane 5 shows the biotinylated protein ladder. The ∼70-kDa bands correspond to NT-biotin. The blot is representative of two similar analyses. (D) DIC images of NT-biotin-coated SVA-beads with or without 10 µM IP_3_. (E) Summary results show means ± SEM, n = 5. *P* = 0.40. Scale bars (A and D) represent 20 µm.

Beads without a surface-coating of biotinylated protein, or beads coated with either biotin-BSA or denatured biotin-NT did not aggregate, whereas beads with immobilized NT aggregated (**Figures 4A and 4B**). Immobilization of denatured NT was confirmed by Western blotting (**Figure 4C**). Although immobilized NT bound IP_3_ appropriately (**Figure 3C**), IP_3_ had no significant effect on the aggregation of NT-beads (**Figures 4D and 4E**). These results suggest that interactions between native NT are sufficient to cause bead aggregation, but the interactions are unaffected by IP_3_ binding under the conditions used for these analyses.

### The suppressor domain of type 1 IP_3_ receptor mediates interactions between N-termini

**Figure 5:**
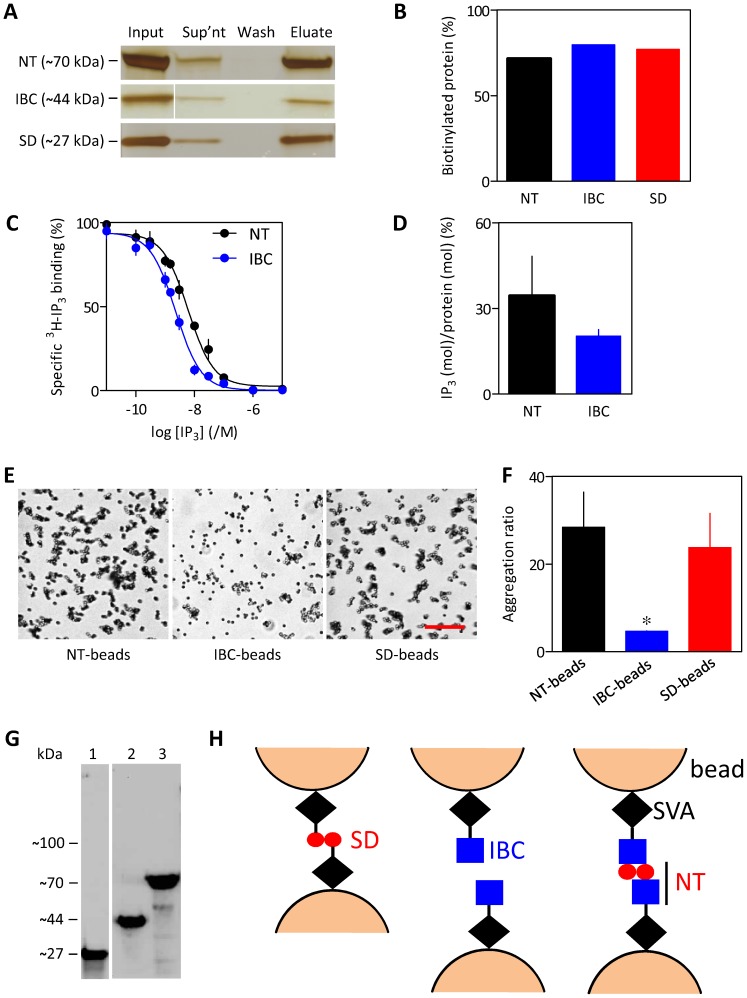
The SD of IP_3_R1 causes aggregation of beads. (A) Silver-stained gel using equivalent amounts of material shows the proteins present in the incubation used to prepare SVA beads (input), the unbound protein (supernatant), the washings from the beads (wash), and the proteins eluted from the beads (eluate, 85°C for 10 min). (B) Summary results show the percentages of protein immobilized on the beads for NT, IBC and SD. (C) Equilibrium competition binding to NT-biotin and IBC-biotin with ^3^H-IP_3_ (0.75 nM). (D) Functional protein quantified from the amount of ^3^H-IP_3_ (mol) bound per mol of protein. Results (C and D) are means ± SEM, n = 3. (E) DIC images of the indicated beads. Scale bar = 20 µm. (F) Summary results (means ± SEM, from 3 independent experiments each with 5 fields). *P = 0.041 relative to NT-beads. (G) Western blot, probed using HRP-conjugated streptavidin, shows purified samples of SD-biotin (lane 1; ∼27 kDa), IBC-biotin (lane 2; ∼44 kDa) and NT-biotin (lane 3; ∼70 kDa). Lane 1 is from a separate blot. The protein preparations did not contain detectable amounts of residual GST-tagged SD, IBC or NT fragments which have predicted sizes of 54 kDa, 71 kDa and 96 kDa, respectively. (H) The SD (left) or NT (right) interact and thereby cause beads to aggregate, whereas beads coated with IBC (centre) do not aggregate.

The suppressor domain (SD, residues 1–223) is essential for IP_3_R activation [20], [21] and binding of IP_3_ to the IP_3_-binding core (IBC, residues 224–604) rearranges the relationship between the IBC and SD [12], [13]. We therefore assessed the interactions between isolated SD and IBC by immobilizing each on SVA beads. The NT, IBC and SD fragments were biotinylated with similar efficiency (**Figures 5A and 5B**). Furthermore, quantitative comparisons of the density of ^3^H-IP_3_ binding sites (B_max_) derived from equilibrium competition binding to the NT and IBC (**Figure 5C**) and quantification of the amounts of biotinylated proteins (**Figure 5A**) established that there was no statistical difference in the stoichiometry of IP_3_ binding (mol IP_3_/mol protein) for the two fragments (**Figure 5D**). These results establish that the fragments used are likely to be similarly immobilized on SVA beads and, at least for the IBC and NT (the only fragments that bind IP_3_), that each is similarly bioactive. Aggregation of SD-beads was similar to that of NT-beads, whereas IBC-beads did not aggregate (**Figures 5E and 5F**). GST can itself dimerize [22] and might therefore have contributed to bead aggregation if residual GST-tagged SD or NT were present in the protein samples after purification. This is unlikely as none of the protein samples contained detectable GST-tagged fragments (**Figure 3A** **and** **Figure 5G**). These results suggest that interactions between the NT are mediated by the SD (**Figure 5H**).

The bead aggregation assay developed in this study is an economical and rapid method for detecting interactions between two ligands that should be generally applicable to analysis of low-affinity interactions between macromolecules. The assay is best suited to qualitative assessment of such interactions and the effects of varying incubation conditions in parallel experiments. We have not attempted to define the absolute detection sensitivity of the assay, but we have shown that it resolves statistically significant differences between different immobilized ligands and that it succeeds in detecting specific interactions between the NT domains of IP_3_R that are too weak to detect by immunoprecipitation [23]. The structure of the NT docked into a low-resolution structure of native IP_3_R [13] indicates that SD-SD interactions within an IP_3_R tetramer are unlikely. Instead, our results suggest that NT domains of IP_3_R may interact in a head-to-head fashion, consistent with electron microscopy [19]. A physiological consequence of such head-to-head interactions may be the formation of ER stacks, which have been suggested to require the NT of IP_3_R1 [18]. Interactions between the NT of IP_3_R may also contribute to clustering of IP_3_R [24], [25], and influence interactions of IP_3_R with modulatory proteins [15], [16].

## Materials and Methods

### Expression and purification of IP_3_ receptor fragments

All IP_3_R fragments lack the S1 splice site, but they are numbered by reference to full-length rat IP_3_R1 containing the S1 splice site (GenBank accession number: GQ233032.1). N-terminal fragments were expressed with an N-terminal glutathione S-transferase (GST) tag followed by a PreScission cleavage site (LEVLFQGPLGS) to facilitate purification, and a C-terminal biotinylation sequence (EFGGGLNDIFEAQKIEWHE) for immobilization on SVA beads (**Figure 2E**). Preparation of N-terminal fragments from constructs in the pGEX-6P2 plasmid (GE Healthcare, Cardiff, UK) was described previously [26]. The sequences of all constructs were verified.

The open reading frames of the tagged SD, IBC or NT were cloned into the expression vector pGEX-6P2 and transformed by heat-shock into AVB101 *E. coli* (Avidity, Aurora, Colorado, USA). These bacteria have an IPTG-inducible birA gene to allow expression of biotin ligase. Bacteria were incubated at 37°C for 16 h on LB-agar containing ampicillin (100 µg/mL). A resistant colony was then grown in 20 mL of ampicillin-containing LB medium in an orbital shaker (250 rpm, 37°C). After 12 h, 10 mL of the culture was diluted into ampicillin-containing LB medium (500 mL) and incubated (150 rpm, 22°C) until the optical density at 600 nm (OD_600_) reached ∼1 (∼9 h). Protein expression was induced by addition of IPTG (0.5 mM), and the pellet (6000x *g*, 20 min) was collected after incubation for 20 h (150 rpm, 15°C). The pellet was washed with phosphate-buffered saline (PBS, 10 mL; 13.7 mM NaCl, 0.27 mM KCl, 1 mM Na_2_HPO_4_, 0.2 mM KH_2_PO_4_, pH 7.3, 4°C) and re-suspended in Tris-EDTA medium (TEM, 44 mL; 50 mM Tris-HCl, 1 mM EDTA, pH 8.3 at 4°C) containing protease inhibitor cocktail (Roche, Hertfordshire, UK; EDTA-free complete protease inhibitor cocktail, 1 tablet/50 mL). PopCulture (Merck Millipore, 10% v/v) and 2-mercaptoethanol (1 mM) were added, and the suspension was incubated with lysozyme (100 µg/mL) and RNase (10 µg/mL) on ice for 30 min. The lyzate was sonicated on ice for 20 s and the supernatant was recovered (30000× *g*, 1 h).

IP_3_R fragments were purified via the N-terminal GST tag. The supernatant (50 mL) was centrifuged to remove debris (6000× *g*, 30 min, 4°C), mixed with glutathione Sepharose 4B beads (1 mL of 50% slurry, GE Healthcare), incubated with gentle end-over-end rotation (22°C, 30 min) and transferred to a PD-10 column (GE Healthcare). All subsequent steps were performed at 4°C. The beads were washed three times with PBS (5 mL) containing protease inhibitor cocktail, and three times with PreScission buffer (5 mL; 50 mM Tris-HCl, 1 mM EDTA, 150 mM NaCl, 1 mM dithiothreitol, pH 7.5). GST-PreScission protease mix (40 µL added to 460 µL of PreScission buffer; GE Healthcare) was then added to the beads. The column was sealed and incubated with gentle end-over-end rotation for ∼4 h. The eluate containing purified biotinylated IBC, SD or NT was then collected and stored at −80°C. GST-tagged PreScission is retained by the glutathione beads.

### Preparation of coated SVA beads and bead aggregation assay

The DNA sequences (Invitrogen) used were adapted from [27] (5′-3′, single-stranded sticky ends are underlined): biotin-dsDNA-F, biotin-ACCCTTCGCACAGTCAATCCAGAGAGCCCTGCCTTTCATTACGATCATAACTTGG and biotin-dsDNA-F′, biotin-ACCCTTCGCACAGTCAATCCAGAGAGCCCTGCCTTTCATTACGACCAAGTTATGA . For analysis of DNA-induced aggregation of beads, streptavidin-coated magnetic T1 Dynal beads (SVA-beads, Invitrogen) were washed three times with TEM (25 µL) using a DynaMag-2 magnet (Invitrogen). The washed beads (10 pmol binding sites, ∼300,000 binding sites/bead) were then incubated with either biotin-dsDNA-F or biotin-dsDNA-F′ (20 pmol) for 30 min at 22°C with gentle rotation. For analyses of interactions between the DNA sequences, equal amounts of the two sets of coated beads were mixed and incubated in TEM (25 µL) for 30 min at 22°C with gentle rotation.

For analyses of interactions between N-terminal fragments of IP_3_R, washed SVA beads (10 pmol binding sites) in 25 µL of TEM were incubated with 20 pmol of NT-biotin, SD-biotin, IBC-biotin, BSA-biotin (Sigma-Aldrich) or denatured NT-biotin (85°C, 10 min) at 22°C with gentle rotation. After 30 min, coated SVA-beads were magnetically isolated, washed twice with TEM (50 µL) and re-suspended in TEM (25 µL). For imaging, SVA-beads were diluted 10-fold in TEM, plated on glass-bottomed imaging dishes (MatTek, number 0 coverglass) and allowed to settle for 15 min. Differential interference contrast (DIC) images were captured using an iXon 887 EMCCD camera (512 pixels×512 pixels; Andor Technology, Belfast, Ireland) and an Olympus IX81 microscope with a x60 objective, and acquired using CellR imaging software (Olympus Europe, Hamburg, Germany).

To quantify amounts of protein immobilized, coated beads (150 pmol or 10 pmol of binding sites in 20 µL for silver stained gel and Western blot, respectively) were heated (85°C, 10 min) and the supernatant was used for SDS PAGE with NuPAGE 4–12% Bis-Tris gels (Invitrogen). Gels were developed using a Pierce silver-stain kit (Thermo Fisher Scientific, Cramlington, UK). For Western blots, proteins were transferred to PVDF membranes using the iBlot system (Invitrogen, Carlsbad, CA) and membranes were blocked by incubation (1 h) with Tris-buffered saline (TBST; 20 mM Tris, 140 mM NaCl, 0.1% v/v Tween 20, pH 7.6) containing BSA (5% w/v). The membrane was rinsed with TBST, incubated with HRP-conjugated streptavidin (1∶4500, Thermo Fisher Scientific, Cramlington, UK) in TBST containing BSA (1% w/v) for 1 h and then washed with TBST (3×5 min). Bands were detected using SuperSignal West Pico chemiluminescent substrate (Thermo Fisher Scientific) and quantified using ImageJ (version 1.44o).

### Analysis of bead aggregation

The protocol for analysis of bead aggregation is summarized in **Figure 2A**. For each image, an intensity threshold was applied, which selects pixels that define beads (**Figure 2C**). Ten single beads were randomly selected from each field and a one pixel-thick outline was used to define the perimeter of each bead, from which its area was calculated (excluding the outline pixels). In subsequent analyses, particles with areas greater than the mean area of a single bead plus twice the standard deviation (the area threshold) were considered as aggregates. The aggregation ratio was then calculated from:


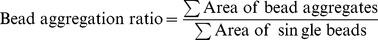


 (**Equation 1**)

For each experiment (n), 5 fields each containing ∼1000 beads were analyzed. Statistical analyses used unpaired Student's *t*-tests (GraphPad Prism, version 5), with statistical significance reported in figure legends.

### ^3^H-IP_3_-binding assay

Equilibrium competition binding assays were performed at 4°C in TEM (500 µL) containing ^3^H-IP_3_ (0.75 nM, 681 GBq/mmol, PerkinElmer Life and Analytical Sciences, Beaconsfield, UK) and either coated SVA beads (1.8 pmol binding sites) or soluble NT-biotin or IBC-biotin (0.51 pmol). After 5 min on ice, incubations were terminated by addition of cold TEM (500 µL) containing polyethylene glycol (PEG) 8000 (30% v/v) and γ-globulin (750 µg). After 5 min on ice, the pellet was isolated by centrifugation (20000x *g*, 5 min), washed (500 µL of 15% PEG in TEM) and dissolved in 2% Triton X-100 (200 µL). Pellets were solubilized by sonication and their radioactivity was determined by liquid scintillation counting in EcoScintA (1 mL, National Diagnostics, Atlanta, GA). Non-specific binding was defined by addition of unlabelled IP_3_ (1 µM). Binding curves were fitted to Hill equations using GraphPad Prism, from which half-maximal inhibitory concentrations (IC_50_) and thereby equilibrium dissociation constants (K_D_) were derived. pK_D_ values (negative logarithm of K_D_) were used for statistical analyses.
